# Introductory Lecture to the Course of Materia Medica in the University of Pennsylvania

**Published:** 1842-11-19

**Authors:** 


### BIBLIOGRAPHICAL NOTICES.

*Introductory Lecture to the course of Materia Medica in the University of of Pennsylvania. Delivered November* 2, 1842. By George B. Wood, M. D. 8vo. pp. 27.

Professor Wood's lecture on "the proper choice of medicines," is an appropriate discourse, polished in style, and judicious in advice. The professor offers a series of hints to the young physician for his guidance in the choice of medicines, which embody views to which all sound practitioners will heartily respond. The professor, in the first place, recommends the inexperienced practitioner, as a general rule, not to go beyond the limits of the officinal catalogue of the national pharmacopœia for his selection of remedies. This caution is to be understood as a check upon the employment of remedies "of newly-asserted or revived pretensions," without properly attested credentials, and is particularly addressed to the practitioners of immature experience.

The professor expresses himself forcibly and justly with regard to the class of physicians who deal in "heroics." We suspect, however, that this style of practice is less characteristic of "the West," at present, than the professor "is led to suppose." The well drawn up reports of cases which fill the pages of the western journals, give evidence of the prevalence of a judicious and *moderate* therapeutics. We quote Dr. Wood's remarks:

"There is a class of practitioners who seem to look upon diseases as the Stoics did upon sins, as all equally heinous. No sooner do they catch a glimpse of something suspicious in the distance, than they conclude at once that it is an enemy, and, without estimating his strength, prepare to crush him by the most energetic measures. In every low black schooner they discover a pirate, and direct their paixan guns indiscriminately against the ship of the line and the cockboat. Happily, this disposition is less prevalent than formerly, and, in our parts, has in great measure left the regular profession to seek a refuge among empirics. In the West, however, we are led to suppose that it still prevails with many practitioners. In that section of our country, they are accustomed to every thing on a grand scale, from their magnificent streams and prairies, their gigantic trees and great men, intellectually as well as physically, to their drachm doses of calomel. Perhaps the diseases, as they assert, may partake of the same gigantic character, and require corresponding treatment. Let us hope that they will hereafter participate in our experience, and, whether from a change in the character of the disease, or in the estimate of its force, learn, as we have done, the sufficiency, in most cases, of milder measures. I would not, however, be considered as advocating an inert treatment of disease. I am in favour neither of the ptysan practice of the older French physicians, nor of the mere moonshine of the Homœopathists. All I mean is, that the character of the medicine and its dose should be regulated by the nature of the disease; that we should treat mild cases by lenient and persuasive measures, and launch our thunders only at the refractory and the violent."

Dr. Wood objects alike to the unnecessary multiplication of heterogeneous ingredients in our formulæ, and to the opposite extreme of too great a simplicity in prescriptions. The tendency of pathology and therapeutics at the present time is in the opposite direction to this extreme simplicity; and Dr. Wood's views are in confirmation of the prevailing opinions.

Dr. Wood dwells strongly upon "the restless love of what is new, which, while it is producing much good, is working also no inconsiderable amount of evil," in medicine as elsewhere. Glancing at the wearisome rapidity of the changes proposed in the nomenclature of chemistry and botany, he cautions his hearers at length not to be led astray by this love of novelty in the choice of medicines. His observations on this point are excellent; the tone of them is, perhaps, imbued with a little too much conservatism:

"All the most valuable and best tried instruments of our art are but too apt to fail in obstinate cases of disease, and even where success is probable or certain in the end, it is too often slow. In our extreme anxiety and impatience, we are ready to catch at any aid that is confidentally held out to us; and, as most new medicines or preparations come recommended by a never failing success in the hands of their introducers, we are not without seemingly reasonable hope of advantage from them. In addition to the mere inducements of novelty, we have the uneasiness under a heavy responsibility, and the fear that we may leave some possible means untried of acquitting ourselves well in the almost fearful charge entrusted to us. Many yield to these inducements, and make an eager trial of the new remedy. Perhaps accident, and those various circumstances which very frequently conspire to produce a false conclusion as to the efficacy of a particular treatment, may work in its favour, and we may thus, from a partial experience, acquire a confidence which may lead to its further and more extensive employment, until the tide of fortune changes, and repeated failures at length conduct us back to a just estimate of its value. The continuance of the same causes leads subsequently to similar results. With each newly proposed remedy we run the same round of promising trial, partial success, and ultimate disappointment; and the consequence sometimes is, that drawn off from established methods of cure in pursuit of these *ignes fatui*, we find ourselves at last unsettled in practice and opinion, distrustful of the old without having acquired confidence in the new, and almost ready to surrender in despair all reliance upon the efficacy of medicine. Of newly proposed remedies, many are mere revivals of those once employed but afterwards neglected or forgotten; while, of the remainder, the great majority are wholly incapable of maintaining the place into which they have been temporarily elevated. Since the period at which I commenced the practice of medicine, iodine and its compounds are almost the only new remedies which have come into general use; for morphia, quinia, strynchnia, creasote, &c., are merely the isolated active principles of medicines before well known; while numerous substances, original or revived, for which high claims were asserted, are either altogether forgotten, or treated as objects rather of curiosity than real service. Let me, therefore, strongly urge you always, in your choice of medicines, to lean decidedly towards those of established reputation. Do not neglect the old tried servants of your professional fathers, for the crowd of younger applicants for your favour, whose only claims are a new face, a good deal of pretension, and a list of recommendations from persons you do not know. But, at the same time, I am far from wishing to confine you to the paths before trodden, and to close the access to something higher than we have yet attained. Well regulated efforts to widen the circle of the useful are highly laudable. What I wish to impress on you is, that you should not adopt a medicine because it is new—that you should, in fact, consider its novelty as a ground of suspicion, and should admit it into your confidence only upon strong and trustworthy recommendation, and after a strict examination into its merits."

We entirely subscribe to the following remarks:

"I have only one other limitation to propose to you in your selection of medicines. Never, under any circumstances, employ those of which the composition is kept secret. Such medicines will be constantly urged upon your notice with the highest pretensions. The preparer will offer them to your acceptance and humbly beg for a trial, either in the hope of subsequently obtaining your recommendation, or at least with the intention of making use of your name. Your patients, yielding to the solicitations of friends, or prompted by their own secret hopes, will press you to permit or authorize their use. But steel yourselves against all such solicitations, and resolve that your hand, at least, shall not be the one to fix a stain upon the fair fame of your profession. You may justly ask the reasons for such a positive rejection of remedies of asserted value. Is it not obvious that, so long as their nature and preparation are concealed, you can have no such certain knowledge of their mode of action as to justify you in their employment to meet any given indication? It may be argued, on the opposite side, that we are equally ignorant of the precise composition of many other well known remedies as they come from the laboratory of Nature; that the secret medicine in question may have been so frequently tried, under every variety of circumstances, that, in relation to its physiological and therapeutical effects, it is as well known as those of a more legitimate character; and that we have no right to reject offered means of relief to our patient, however irregular these means may be. But the answer is clear; that a substance produced by nature, even though its composition may not be known, can always be relied on as identical, if obtained under similar circumstances, and treated in the same manner; while, in relation to the secret medicine, you can have no such confidence, as its mode of preparation depends on the caprice or varying views of an individual not always of the best character; and, even though one parcel of it may have been profitably employed under certain circumstances, you can have no satisfactory proof that another parcel will have the same effect. But there are other and higher grounds for your utter rejection of such medicines. By allowing yourselves to be drawn into their use, you would give to unprincipled men the opportunity of citing your example as a rule for others. No matter how careful you may be, in employing the nostrum, to confine it within perfectly safe limits—no sooner will you have touched the vile thing, than the fact will be proclaimed wherever your name has influence; you will be emblazoned in advertisements, and heralded in placards as its indiscriminate patron, and will thus, even against your wishes, be made an instrument for extending its general reputation, and establishing it in the public confidence. After this, it will be in vain that you may disclaim your asserted favour. You cannot but acknowledge that you have used it; and all else that you may say will be ascribed to professional or personal jealousy, and will tend still further to benefit the empiric, by the opportunity it will afford him of exhibiting himself to the public as a persecuted man.

Under these circumstances, would not your first inconsiderate step be answerable for a proportion of all the mischief which may arise from the misapplication of the medicine? Would you not, moreover, be lending your countenance to the general cause of empiricism? Would not the whole rabble of quacks shout out your name as one of their supporters? And would not your profession itself be in some measure degraded by this association of one of its members with such a cause? It is highly important, therefore, to keep yourselves strictly within the regular limits. Exceed these, even though in a slight degree, and you lose all control over the result. You cannot calculate the evils which may flow from one false step. What is any possible advantage which may accrue in a single case in which you might be disposed to employ the nostrum, compared with all this general evil? I do not speak thus as a mere matter of course, but with a strong sense of the duties of our high calling, and of the imperative obligation of every member of the profession to avoid doing anything which might degrade its character, and limit its sphere of usefulness. I beseech you, gentlemen, as you regard this character, as you value your own reputation and future comfort, to keep yourselves clean from every taint of empiricism. Of what consequence is a little pecuniary profit; nay, of what consequence are heaps of gold acquired by such imposture? Does not a feeling of disgrace cleave to their possessor through his whole future life? Does not the finger of scorn point to him while he lives, and at his death does he not leave an inheritance of shame to his descendants, so that his son and his son's son must blush at the mention of his occupation? I presume, gentlemen, there is not one among you who would not rather be the offspring of the humblest wood-chopper or sweeper of the streets, if an honest man, than of the most prosperous quack who ever revelled in wealth, purchased by a base course of deception, and at the cost of injury to thousands. You would shrink, of course, from leaving to those who may come after you a legacy which you would look upon as a disgrace from one of your own predecessors. But I have been led away from the point to which I wished especially to direct your attention. There is no danger of your becoming quacks—there may be some, that, unless carefully on your guard, you may afford that degree of countenance to quackery which is implied in the occasional employment of secret nostrums. Let me again urge upon you, even at the possible chance of losing a temporary advantage, to shun them altogether, and, so far as your influence may extend, to discourage their use by others of your professional brethren."

